# Development of enhancer-trapping and -detection vectors mediated by the *Tol2* transposon in zebrafish

**DOI:** 10.7717/peerj.6862

**Published:** 2019-04-30

**Authors:** Shuheng Chan, Dan Shen, Yatong Sang, Saisai Wang, Yali Wang, Cai Chen, Bo Gao, Chengyi Song

**Affiliations:** Yangzhou University, Institute of Animal Mobilome and Genome, College of Animal Science & Technology, Yangzhou, Jiangsu, China

**Keywords:** Enhancer trapping, Enhancer activity, Tol2, Zebrafish

## Abstract

Enhancers are key transcriptional drivers of gene expression. The identification of enhancers in the genome is central for understanding gene-expression programs. Although transposon-mediated enhancer trapping (ET) is a powerful approach to the identification of enhancers in zebrafish, its efficiency varies considerably. To improve the ET efficiency, we constructed *Tol2*-mediated ET vectors with a reporter gene (*mCherry*) expression box driven by four minimal promoters (Gata, Myc, Krt4 and Oct4), respectively. The ET efficiency and expression background were compared among the four promoters by zebrafish embryo injection at the one-cell stage. The results showed that the Gata minimal promoter yielded the lowest basic expression and the second-highest trapping efficiency (44.6% at 12 hpf (hour post-fertilization) and 23.1% at 72 hpf, *n* = 305 and *n* = 307). The Krt4 promoter had the highest trapping efficiency (64% at 12 hpf and 67.1% at 72 hpf, *n* = 302 and *n* = 301) and the strongest basic expression. To detect enhancer activity, chicken 5′HS4 double insulators were cloned into the two ET vectors with the Gata or Krt4 minimal promoter, flanking the *mCherry* expression box. The resulting detection vectors were injected into zebrafish embryos. *mCherry* expression driven by the Gata promoter (about 5%, *n* = 301) was decreased significantly compared with that observed for embryos injected with the ET vectors (23% at 72 hpf, *n* = 308). These results suggest that the insulators block the genome-position effects and that this vector is fit for enhancer-activity evaluation. To assess the compatibility between the enhancers and the minimal promoters, four enhancers (CNS1, Z48, Hand2 and Hs769) were cloned upstream of the Gata or Beta-globin minimal promoter in the enhancer-activity-detection vectors. The resulting recombinant vectors were assayed by zebrafish embryo injection. We found that Z48 and CNS1 responded to the Gata minimal promoter, and that Hand2 only responded to the Beta-globin minimal promoter. In contrast, Hs769 did not respond to either the Gata or Beta-globin minimal promoters. These results suggest the existence of compatibility between enhancers and minimal promoters. This study represents a systematic approach to the discovery of optional ET and enhancer-detection vectors. We are eager to provide a superior tool for understanding functional genomics.

## Introduction

Enhancers are among the most important cis-regulatory elements that play a major role in cell-type-specific gene expression (Guenther et al., 2007; Heintzman et al., 2009), which widely reflects developmental patterning (Sagai et al., 2005) or human genetic disease (Grosveld et al., 1987). Enhancers recruit transcription factors and the transcriptional apparatus to activate their target genes (Smith & Shilatifard, 2014; Ren & Yue, 2015) and can be larger than one megabase (Mb) (Fraser & Engel, 2006). To date, many methods have been developed to discover enhancers, such as enhancer trapping (ET) (Kawakami et al., 2004; Balciunas et al., 2006; Scott et al., 2007; Asakawa et al., 2008), retrovirus-based technologies (Ellingsen et al., 2005; Laplante et al., 2006) and the chromatin immuno precipitation sequencing technique (Mcaninch & Thomas, 2014; Bu et al., 2017). Generally, a basic ET construct is composed of a reporter gene under the control of a minimal promoter (Bellen et al., 1989). Once integrated into the genome, enhancers in the chromosome cannot drive the expression of the reporter gene of the trapping construct unless they induce the activity of a minimal promoter (Bellen, 1999). The rapid development of ET technology has helped identify a large number of enhancers (Balciunas et al., 2004; Liu et al., 2015; Sang et al., 2018). A total of 11 enhancer-detection lines were characterized by *Minos* transposon-mediated enhancer detection screening in *Ciona* (Yoshida & Sasakura, 2012). Transgenic mice with lentiviral vectors carrying single-copy enhancer-detector probes encoding either the marker gene *lacZ* or Cre recombinase were generated and used as an enhancer-detection strategy in mammals (Kelsch, Stolfi & Lois, 2012). Among these methods, transposon-mediated ET represents the most effective insertion in vertebrate systems and has been developed as a gene-delivery tool for gene therapy and insertional mutagenesis (Kebriaei et al., 2017). For instance, it has been applied successfully in medaka (Grabher et al., 2003), zebrafish (Balciunas et al., 2004; Scott et al., 2007; Liu et al., 2015), mouse (Choi et al., 2018) and insect (Koelzer, KöLsch & Panfilio, 2014) models. Transposon-driven vectors generally harbor a reporter-protein-encoding region downstream of a minimal promoter, which is flanked by transposon inverted terminal repeats (Bier et al., 1989; Wilson et al., 1989; Dunne et al., 2014). It seems that the choices of minimal promoter and transposon are equally important for ET efficiency. The *Sleeping Beauty*, *PiggyBac* and *Tol2* transposons are widely used in genetic research using animal models (Kawakami et al., 2004; Balciunas et al., 2006; Scott et al., 2007; Asakawa et al., 2008). Among them, *Tol2* has the highest transgenic efficiency in zebrafish (Shen et al., 2018); thus, we chose Tol2 as the optimized candidate for ET. However, the type of minimal promoter that should be used in ET vectors to achieve a higher efficiency remains an urgent issue. The Krt4 and Gata minimal promoters from zebrafish have been applied in ET technology (Bessa et al., 2009; Chatterjee et al., 2010; Ogura et al., 2009; Trinh & Fraser, 2013). These two minimal promoters can drive green fluorescent protein (*GFP*) expression in transgenic zebrafish, which is fit for ET technology. We were eager to apply other minimal promoters in zebrafish; therefore, we selected an additional two minimal promoters, Myc and Oct4, which are derived from the mouse and are rarely used in zebrafish. To develop an efficient ET vector, we compared the basic expression (*mCherry* expression driven by the minimal promoter itself in the absence of an enhancer) and ET efficiency of four minimal promoters (Myc, Oct4, Krt4 and Gata) in zebrafish, which is an important model organism for the efficient detection of enhancer activity in vivo (Haffter et al., 1996) and a superior model compared with mouse models in large-scale screens. An insulator is a type of DNA element that can protect genes from nearby enhancers or silencers. According to previous studies, a 250 bp “core” containing the 5′HS4 element was effective in blocking enhancer activity (Recillas-Targa et al., 2002), and two copies of the core element functioned as a strong insulator when placed between the enhancer and the promoter (Eissenberg & Elgin, 1991; Chung, Bell & Felsenfeld, 1997). To detect the activity of enhancers efficiently and avoid the effect of the host genomic regulators surrounding them, 5′HS4 insulators were used to flank the *mCherry* reporter gene expression box, which is driven by an enhancer and a minimal promoter. We also evaluated the compatibility between minimal promoters and enhancers. To verify enhancer activity, here, we report an approach that combined ET, insulators and transposons. We demonstrated the feasibility of using this approach to trap active enhancers and attempted to detect their activity.

## Materials and Methods

### Enhancer-trapping vectors

To construct ET vectors, the minimal promoters of Myc (Lovén et al., 2013), Oct4 (Yokota et al., 2016), Krt4 (Chatterjee et al., 2010) and Gata (Bessa et al., 2009) were cloned by high-fidelity PCR from the mouse or zebrafish genome using the primers listed in Table 1. Subsequently, the correct promoters were sub-cloned upstream of the *mCherry* reporter gene in the pTol2-*mCherry* vector using the B*amH*l and A*ge*l restriction sites, respectively. The resulting trapping vectors were named p*Tol2*-Myc-*mCherry*, p*Tol2*-Oct4-*mCherry*, p*Tol2*-Krt4-*mCherry* and p*Tol2*-Gata-*mCherry*.

**Table 1:** Primers used for the cloning of the elements.

Genes	From 5′ to 3′ (the *italicized* text indicates the restriction enzyme cutting sites)	Products size (bp)
MycF	*ACCGGT*TCGCTCCCTCTGCCTCTCGC	510
MycR	*GGATCC*AGATCTCTGCTACGGAGGAGCAGCAG	
Oct4F	*ACCGGT*GCAGTGCCAACAGGCTTTGT	368
Oct4R	*GGATCC*AGATCTGGGGAAGGTGGGCACCCCGA	
Krt4F	*ACCGGT*GTGTGTGTGTGAGAGCAGTC	168
Krt4R	*GGATCC*AGATCTAGGTACGAGAGTGCTCTCTG	
GataF	*ACCGGT*TATTCATTAATAGAATAGAG	1,043
GataR	*GGATCC*CTCAAGTGTCCGCGCTTAGAA	
InsF	*AGATCTGAATTC*GAGCTCACGGGGACAGCCCC	268
InsR	*AGATCTGAATTC*AAGCTTTTTCCCCGTATCCC	
Z48F	GG*GAATTC*GCTCTCGCAGTTGTGGGC	602
Z48R	CC*ACCGGT*CCCCCTGCTTAAGACACAG	
Hand2F	GG*GAATTC*TCACGTTTTCATAAATTCTGAT	295
Hand2R	CC*ACCGGT*GTGTTGTGTGTGGGGTTCAG	
Hs769F	GG*GATATCC*ACCATTTACCACTGCATCGTTCTGG	1,001
Hs769R	CC*ACCGGT*GTGCAAGTGGTCATACCTGTTT	
CNS1F	*GAATTC*GGAATAAAAGAAAAAGCAAAGC	1,013
CNS1R	*ACCGGT*TCTCTCCATCCCCTTTAG	
Beta-globinF	*ACCGGT*GCACTACTAAGCTTCTCGAGGCTAGCTCGCGAGGGCATAAAAGTCAGGGCAGAGCCATCTATTGCTTACATTTGCTTCTGACA	69
Beta-globinR	*GGATCC*ATGAATTCTGTCAGAAGCAAATGTAAGCAATAGATGGCTCTG	

### Enhancer-detection vector

According to Chung, Bell & Felsenfeld (1997), the 5′HS4 insulator, which contains two sequentially connected insulator sequences, was cloned from the chicken genome by high-fidelity PCR using the primers InsF and InsR (Table 1). Then, the 5′HS4 element was inserted upstream and downstream of the *mCherry* expression box in the ET vectors described above, and the resulting enhancer-detection vectors were named pEDV-Gata and pEDV-Krt4.

Four enhancers (Z48, Hand2, CNS1 from the zebrafish genome and Hs769 from the human genome) were cloned by high-fidelity PCR using primers designed according to the GenBank sequences (Table 1).The correct enhancers were sub-cloned upstream of the minimal promoter in pEDV-Gata or pEDV-Krt4 using the E*coR*l and A*ge*l restriction sites.

The mouse Beta-globin minimal promoter was cloned by extending PCR using primers designed according to Carter et al. (2002). This promoter was then inserted upstream of the *GFP* reporter gene, to replace the CAG promoter in the pCAG-*GFP* vector (11150; Addgene, Watertown, MA, USA). The *GFP* expression box driven by the Beta-globin minimal promoter was flanked by the 5′HS4 insulators, to generate another enhancer-detection vector named pEDV-Beta-globin-*GFP*.

### Zebrafish husbandry

The Tubingen strain of zebrafish (*Danio rerio*) was reared according to the standard protocols of the China Zebrafish Resource Center. The zebrafish were maintained at 28 °C (27 ± 2 °C) on a cycle of 14 h light, 10 h dark. Fishes were fed twice a day: once with shelling brine shrimp eggs (in the morning) and once with live adult brine shrimps (in the early evening). This protocol was approved by the Animal Experiment Ethics Committee of Yangzhou University.

### Microinjection of zebrafish

Circular *Tol2* donor constructs (20 pg/nL) were mixed with *Tol2* mRNA (30 pg/nL). The mixture was injected into zebrafish embryos at the one-cell stage. Circular Tol2 donor constructs alone, without the Tol2 mRNA, were injected as the control group. At least 300 live embryos were annotated up to 72 h post-fertilization (hpf) in each group. The expression of the *mCherry* or *GFP* protein was assessed at 12 and 72 hpf using a stereo fluorescence microscope (M165FC; Leica, Wetzlar, Germany).

## Results

### Optimizing the minimal promoter of ET vectors

**Figure 1:**
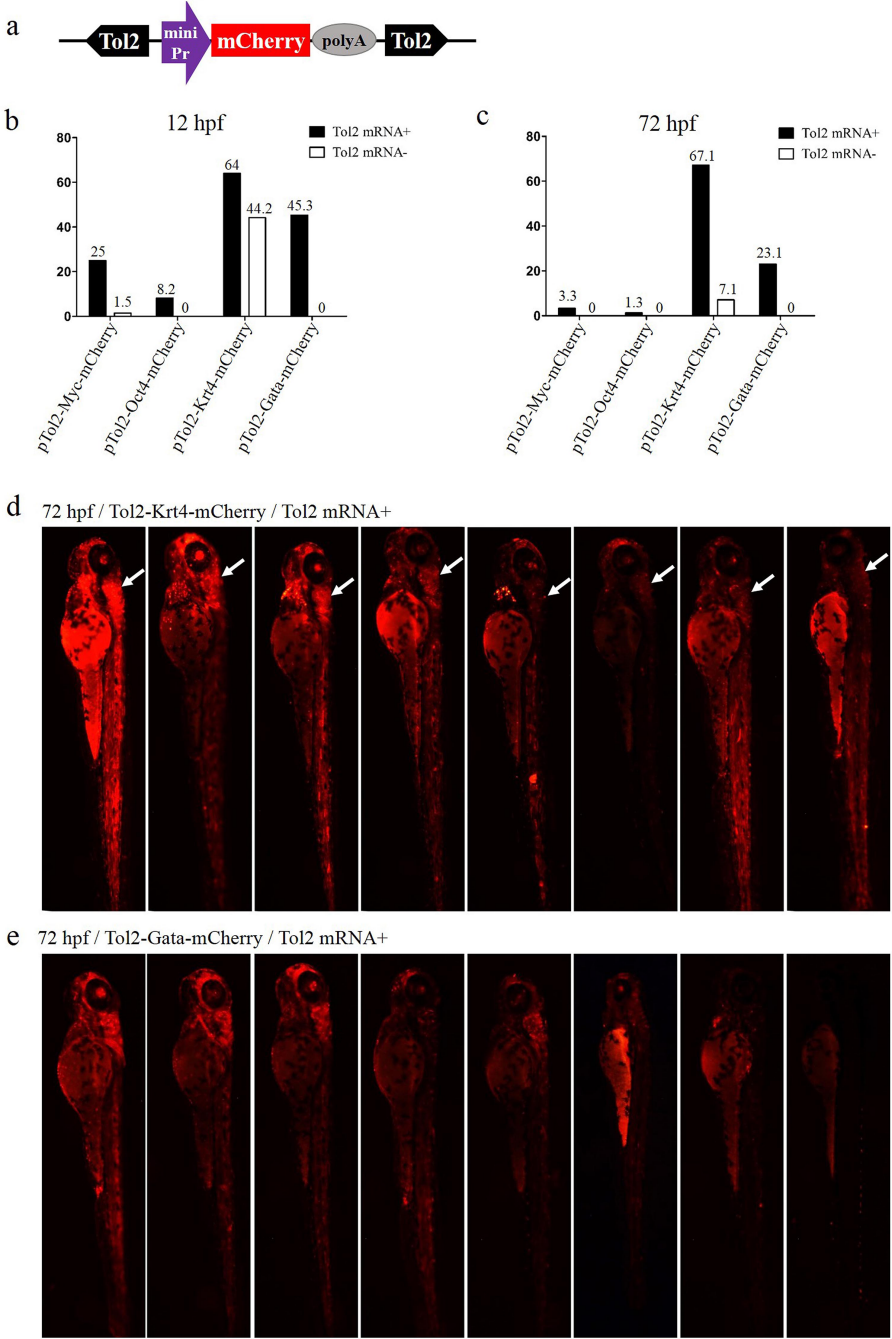
Comparison of the enhancer-trapping efficiency of four minimal promoters in zebrafish embryos. (A) Diagram of the ET vector. The frame contains an ET box flanked by *Tol2* TIRs. The ET box contained a minimal promoter, the *mCherry* ORF and the SV40 polyA. The purple arrow represents the four minimal promoters (Myc, Oct4, Krt4 and Gata), respectively. (B and C) Proportion of *mCherry*-expression-positive embryos injected with ET vectors at 12 and 72 hpf. Embryos at the one-cell stage were co-injected with ET plasmids (20 pg/nL) mixed with the *Tol2* mRNA (30 pg/nL) or were injected with enhancer plasmids (20 pg/nL) alone. (D) *mCherry* expression in embryos injected with the ET vector carrying the Krt4 minimal promoter at 72 hpf; the white arrow denotes the re-occurring pattern detected in the cerebellum. (E) *mCherry* expression in embryos injected with the ET vector carrying the Gata minimal promoter at 72 hpf.

To determine the optimal minimal promoter for the ET vector, four *Tol2*-mediated ET vectors were constructed using the Myc, Oct4, Krt4 and Gata minimal promoters to drive the *mCherry* ORF (Fig. 1A), respectively. These vectors were injected with or without *Tol2* transposase mRNA into zebrafish embryos, to check ET efficiency. The rate of positive embryos (carrying the *mCherry* signal) at 12 or 72 hpf was the highest in the Krt4 group, followed by the Gata, Myc and Oct4 groups (Figs. 1B and 1C). However, the basic expression level was also the highest in the Krt4 group compared with the remaining three groups, which suggests that the Krt4 minimal promoter itself drives *mCherry*, resulting in a high background expression that disturbs ET (Trinh & Fraser, 2013). The rate of *mCherry*-positive embryos at 12 or 72 hpf was second highest in the Gata group, while the basic expression noise was negligible in this group, which suggests that the Gata minimal promoter can be used in ET. Furthermore, our results showed reoccurring patterns in the cerebellum of embryos injected with the Krt4 group (Fig. 1D), which indicated that Krt4 may remain active as a strong promoter (Bellen, 1999), as opposed to what was observed for the Gata group (Fig. 1E). In the Myc or Oct4 group, the rate of positive embryos was very low compared with the Krt4 and Gata groups.

### Minimizing position effects in pEDVs using insulators

**Figure 2:**
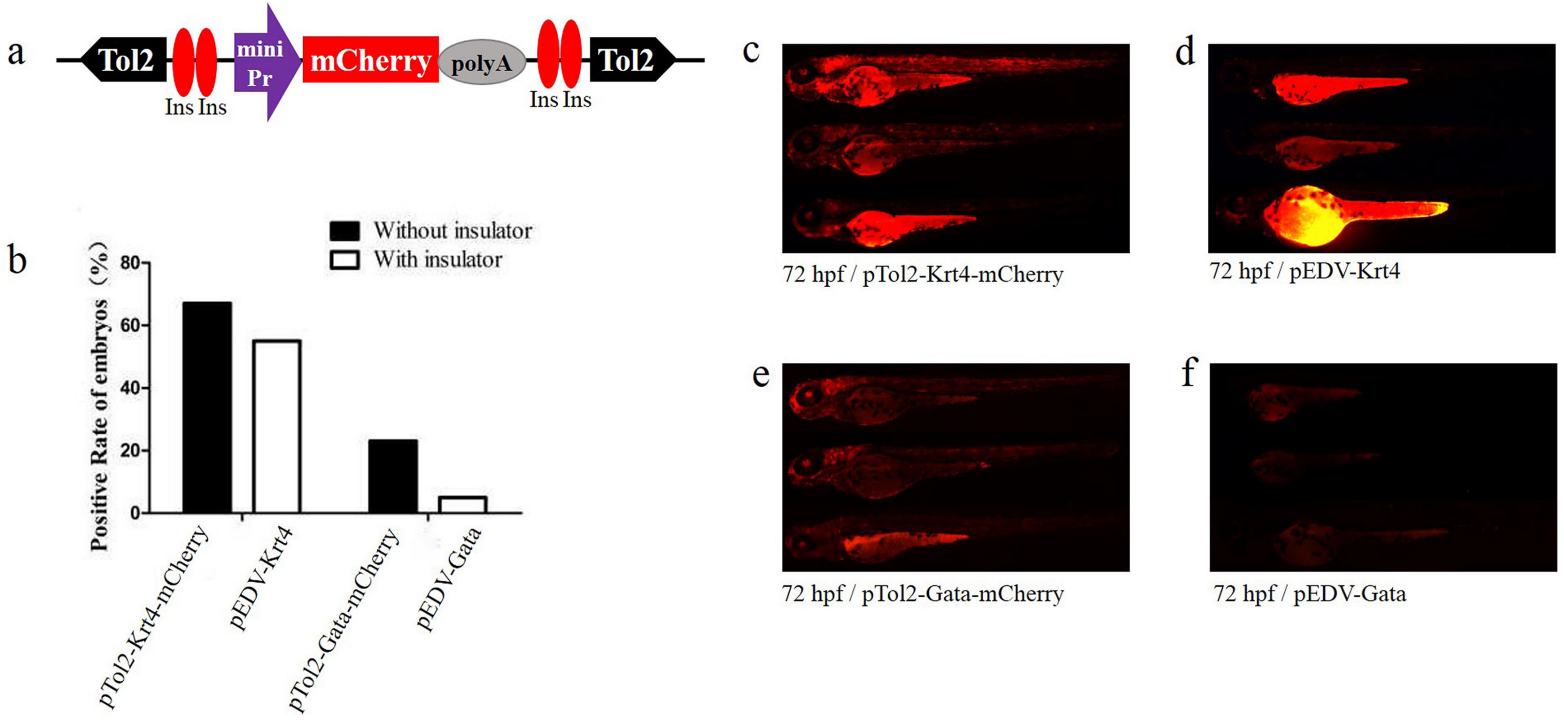
Functional assay of the chicken “5′HS4” insulators in the enhancer-detection vector. (A) Diagram of the zebrafish enhancer detection vector (pEDV). The frame contains two copies of the chicken “5′HS4” insulators (Ins, shown in red ovals), which flanked the ET box. (B) *mCherry* expression rate among embryos injected with the four vectors at 72 hpf: two vectors that included insulators (pEDV-Krt4 and pEDV-Gata) and two vectors that did not include insulators (pTol2-Krt4-*mCherry* and pTol2-Gata-*mCherry*). (C–F) *mCherry* expression in the embryos injected with the various vectors.

To verify the newly identified enhancer, we constructed detection vectors by introducing two 5′HS4 insulators based on the ET vectors pEDV-Gata and pEDV-Krt4 (Fig. 2A). The results showed that the *mCherry* expression rate (∼5%, *n* = 301) in embryos injected with pEDV-Gata was decreased significantly compared with that detected in embryos injected with p*Tol2*-Gata-*mCherry* (23%, *n* = 308) (Fig. 2B). In addition, embryos injected with pEDV-Gata did not show any expression patterns compared with the multiple expression patterns detected in embryos injected with p*Tol2*-Gata-*mCherry* (Figs. 1E, 2E and 2F). This indicates that the 5′HS4 insulators blocked the genome position effects and can be used for the identification of enhancer activity (Figs. 2B and 2F). However, the rate of *mCherry* positivity among embryos injected with pEDV-Krt4 was over 55% (*n* = 301), which was slightly lower than that observed for the embryos injected with p*Tol2*-Krt4-*mCherry* (67%, *n* = 302) (Fig. 2B). The expression of *mCherry* was confirmed in the heart, eye and telencephalon tissues of embryos injected with pEDV-Krt4 (Fig. 2D). Taken together, these results suggest that Gata is superior to Krt4 for ET or enhancer detection.

### Compatibility of minimal promoters and enhancers

**Figure 3:**
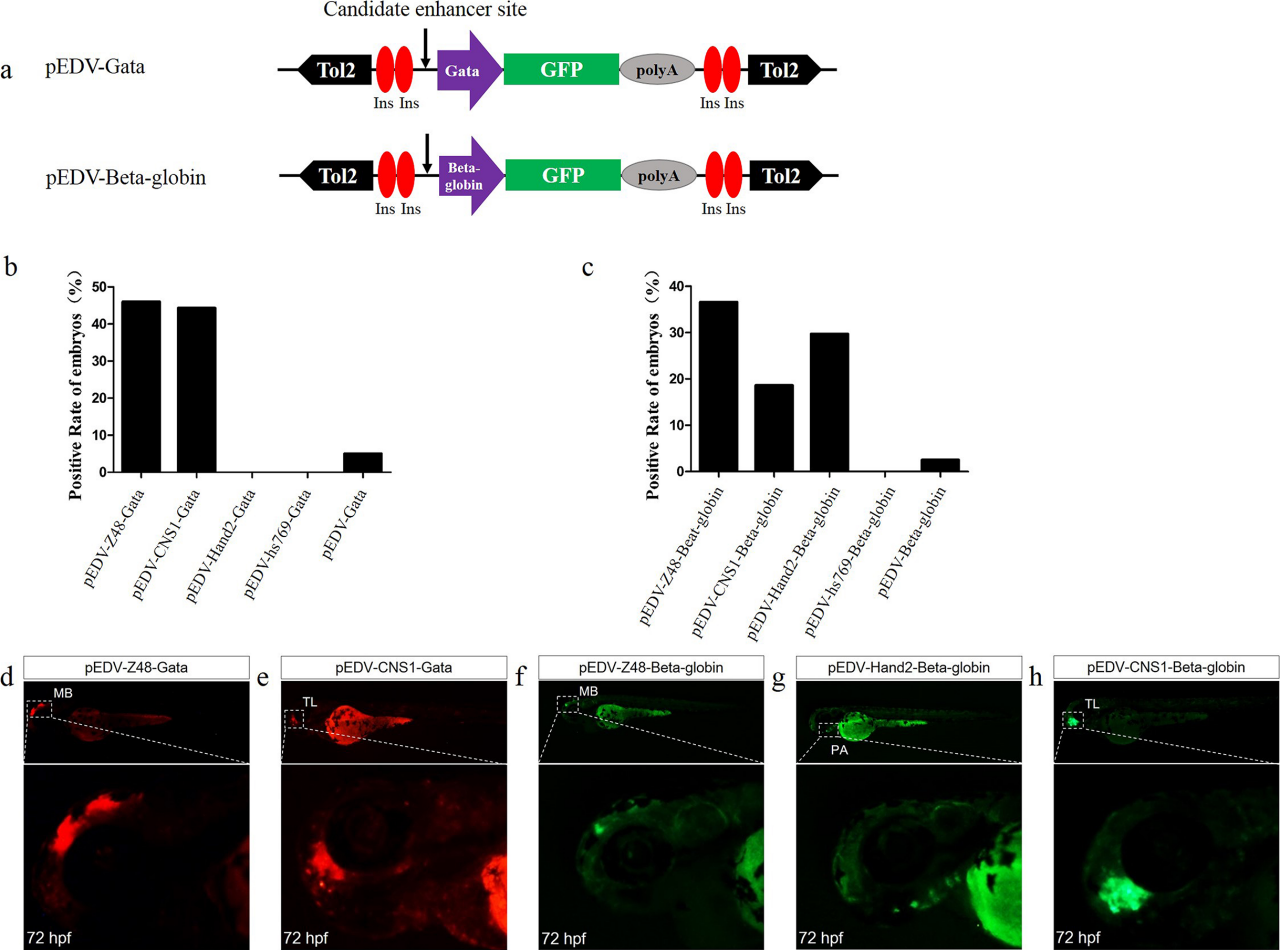
Capacity of the Gata and Beta-globin minimal promoters to respond to different enhancers. (A) Diagram of the enhancer-detection vectors based on the Gata or Beta-globin minimal promoter. (B) *mCherry* expression rate in embryos injected at 72 hpf with the Gata minimal promoter carrying four enhancers (Z48, Hand2, Hs769 and CNS1) or no enhancer. (C) *GFP* expression rate in embryos injected at 72 hpf with Beta-globin carrying three enhancers (Z48, Hand2 and Hs769) or no enhancer. (D and E) *mCherry* expression in the midbrain (MB) and telencephalon (TL) of embryos. (F–H) *GFP* expression in the midbrain (MB), pharyngeal arch (PA) and telencephalon (TL) of embryos.

**Figure 4:**
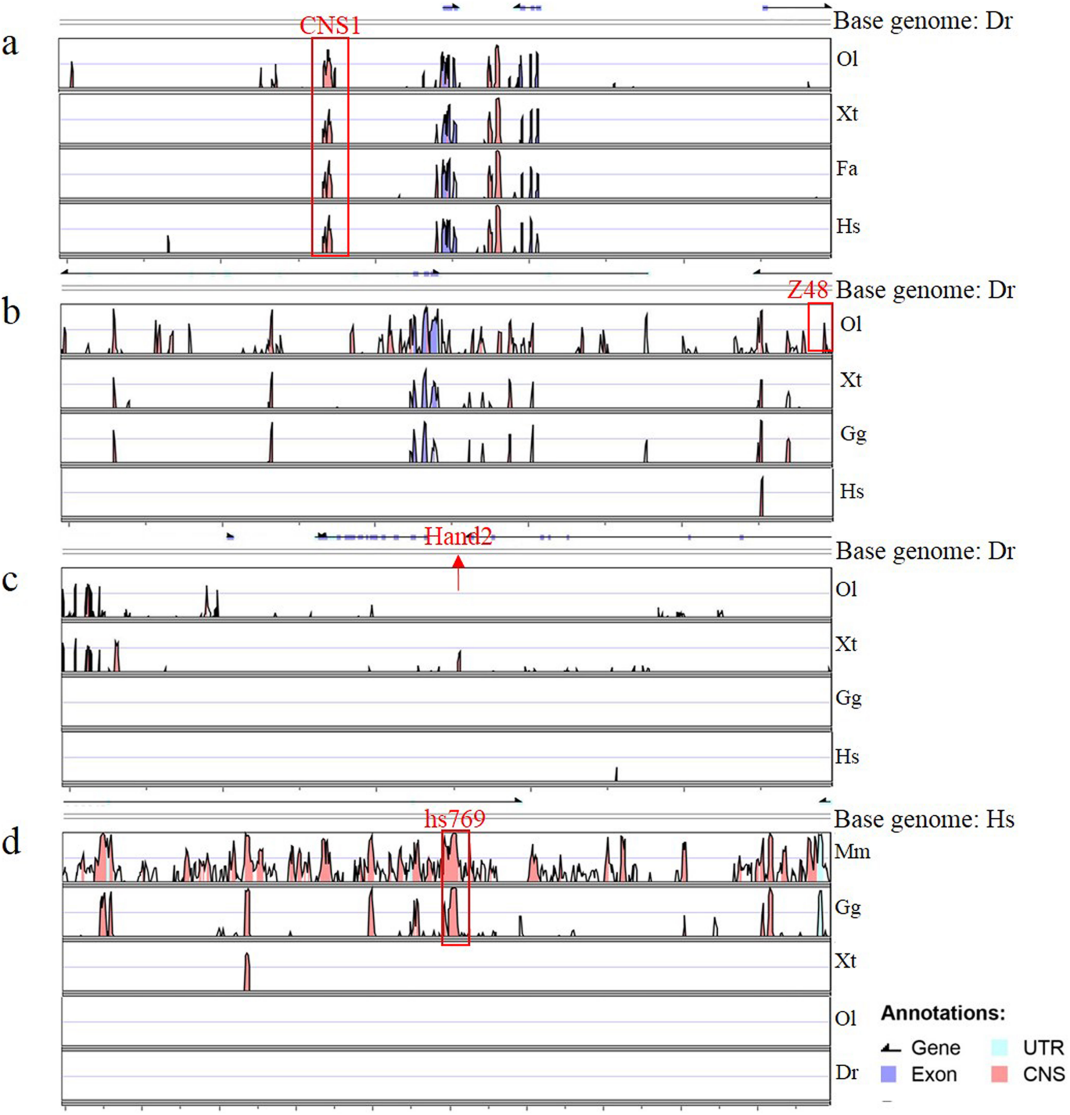
Annotation of four enhancers within 100 kb. (A–D) The conservation of four enhancers in different species. Genomic sequences from *Danio rerio* (Dr), *Oryzias latipes* (Ol), *Xenopus tropicalis* (Xt), *Ficedula albicollis* (Fa), *Gallus gallus* (Gg), *Homo sapiens* (Hs) and *Mus musculus* (Mm) were analyzed in the VISTA browser (http://genome.lbl.gov/vista/mvista/submit.shtml). The red frame indicates a conservation identity >70% and a conservation width >100 bp. The red arrow indicates the location of Hand2 in the zebrafish genome.

To determine whether Gata is compatible with different enhancers, enhancers identified previously (Charité et al., 2001; De La Calle-Mustienes et al., 2005; Bessa et al., 2009; Iklé, Artinger & Clouthier, 2012; MacDonald et al., 2013), CNS1, Z48, Hand2 and Hs769, were subcloned upstream of Gata, and the resulting enhancer-detection vectors were named pEDV-Gata-CNS1, pEDV-Gata-Z48, pEDV-Gata-Hand2 and pEDV-Gata-Hs769, respectively (Fig. 3A). Using a bioinformatics approach, we found that CNS1 was conserved in vertebrates (Fig. 4A), Z48 was conserved in fishes (Fig. 4B), Hand2 was only identified in zebrafish (Fig. 4C) and Hs769 was conserved in mammals and chicken (Fig. 4D). Moreover, the functions of these enhancers, with the exception of Hs769, were verified previously (Bessa et al., 2009; Iklé, Artinger & Clouthier, 2012; MacDonald et al., 2013). Subsequently, each donor plasmid was co-injected with the *Tol2* mRNA into one-cell-stage embryos. We found that embryos that were injected with both pEDV-Gata-Z48 and pEDV-Gata-CNS1 generated distinct *mCherry* expression profiles at 72 hpf, with an *mCherry* positivity rate in embryos of about 45% (*n* = 308). In contrast, embryos that were injected with pEDV-Gata-Hand2 and pEDV-Gata-Hs769 did not express the *mCherry* gene (Fig. 3B), which suggests that neither Hand2 nor Hs769 can activate the Gata minimal promoter. However, the enhancer activity of Hand2 has been confirmed (Iklé, Artinger & Clouthier, 2012). To verify this, the Gata minimal promoter was replaced with the Beta-globin minimal promoter, and the *mCherry* gene was replaced by the gene encoding the *GFP*. We found that Z48, Hand2 and CNS1 regulated β-globin to drive *GFP* gene expression (Fig. 3C), and that the expression patterns were similar to those reported in previous studies (Figs. 3F–3H). However, Hs769 did not regulate *GFP* expression (Fig. 3C). These results suggest the presence of a compatibility problem between enhancers and minimal promoters; thus, additional minimal promoters should be considered for enhancer-activity testing.

## Discussion

Enhancer trapping is an effective technique that can be used to characterize enhancers that regulate the spatiotemporal expression patterns of genes in cells. ET mediated by transposons, which was established in the fruit fly *Drosophila melanogaster*, is an efficient technique for enhancer identification (Liu et al., 2015). ET vectors have two key features: (1) the vector can be inserted into the host genome efficiently and (2) the minimal promoter can maintain a low basic background/noise ratio (Parinov et al., 2004). To construct an efficient ET vector, we used the *Tol2* transposon to mediate the minimal promoter expression cassette. Our results suggested that the *Tol2* transposon significantly increased the integration rate of vectors. To obtain an ideal minimal promoter with a low background expression, we compared four minimal promoters, namely Myc, Oct4, Krt4 and Gata. We found that the expression noise of Gata was almost negligible, and that its ET efficiency was significantly higher than that of Myc and Oct4. The *mCherry* positivity rate and the expression background were the highest for Krt4 compared with the remaining promoters, even in the absence of the *Tol2* mRNA. This indicated that Krt4 itself can drive gene expression without the activation of enhancers, which may disturb the identification of enhancers in the genome (Bellen, 1999). Gata (isolated from zebrafish) was more sensitive to nearby enhancers in the zebrafish genome than were Myc and Oct4 (isolated from mice). Thus, we assumed that the zebrafish Gata minimal promoter was ideal for ET in zebrafish. Therefore, we used the Gata minimal promoter to assemble the enhancer-detection vector. Because the minimal promoter can be affected by enhancers or silencers in genomes (Chung, Whiteley & Felsenfeld, 1993), we used the 5′HS4 insulator (from chicken) in the detection vector, to block the activity of nearby cis-regulatory elements. Our results showed that 5′HS4 shielded the minimal promoter from genome position effects. Moreover, the 5′HS4 insulator worked well in zebrafish, even though it was isolated from the chicken genome and is not conserved in zebrafish. Our results supported the hypothesis that the 5′HS4 insulator may play a crucial role in minimizing position effects in different species. This was also demonstrated by Bessa et al. (2009). In this study, we found that Krt4 drove the expression of a reporter gene by itself, even when shielded with insulators, indicating that the Krt4 minimal promoter might be a strong promoter that is not suitable for enhancer detection.

Here, we constructed two zebrafish enhancer-detection vectors containing 5′cHS4 insulators, the Gata or Beta-globin minimal promoter and the *GFP* or *mCherry* reporter gene. To test the capacity of the promoter to respond to enhancers, four enhancers (CNS1, Z48, Hand2 and Hs769) were cloned upstream of Gata or Beta-globin. We found that Gata was sensitive to CNS1 and Z48, but not to Hand2 and Hs769, while Beta-globin was sensitive to CNS1, Z48 and Hand2, but not to Hs769. CNS1 was conserved in vertebrates, including fishes, amphibians, birds and mammals, and the similarity of the sequence was verified by others in previous studies (MacDonald et al., 2010, 2013). CNS1 was expressed in the telencephalon (Figs. 3E and 3H), which agreed with our previous studies (Shen et al., 2018). Z48 and Hand2 were cloned from zebrafish, Z48 was conserved in fish and Hand2 was only identified in zebrafish. Z48 drove a strong expression in the midbrain (De La Calle-Mustienes et al., 2005; Bessa et al., 2009) and Hand2 promoted expression in the pharyngeal arch in zebrafish (Charité et al., 2001; Iklé, Artinger & Clouthier, 2012). Similar expression patterns were detected here (Figs. 3D, 3F and 3G). Interestingly, Hs769 promoted expression in the neural tube in mice and was conserved in mammals and chicken. However, Hs769 had no effect on the Gata or Beta-globin minimal promoter. These results suggest that the sensitivity of the different promoters to enhancers varied. They also indicate the existence of a compatibility problem between enhancers and minimal promoters and highlight the possibility that the interactions between enhancers and promoters are somewhat specific (Kelsch, Stolfi & Lois, 2012; Rickels & Shilatifard, 2018). A previous study showed that a lentiviral enhancer probe containing a different minimal promoter (hsp68 and thylmp) produced recombination patterns as a result of the random integration of the probe into different genomic loci; those results suggested the presence of interactions between the promoter in the probe and the enhancer in the genomic DNA. Obviously, it is important to obtain appropriate minimal promoters that can reflect enhancer activity.

In summary, we attempted to provide a useful method for the rapid, large-scale genomic screening of enhancers and for the efficient detection of enhancer activity in zebrafish, which will accelerate our understanding of functional genomics.

## Conclusions

We constructed a valuable enhancer-trapping vector, pTol2-Gata-*mCherry*, to efficiently explore additional enhancers in zebrafish. Moreover, we developed enhancer-detection vector successfully to identify enhancer activity. Also, we found the compatibility of minimal promoters and enhancers. These data here represent a systematic approach to the discovery and verification of enhancers.

## Supplemental Information

10.7717/peerj.6862/supp-1 Supplemental Information 1 Raw data for the positive rate of embryos. Positive rate of embryos injected with different vectors under different conditions

## Additional Information and Declarations

### Data Availability

The following information was supplied regarding data availability: The raw measurements are available in File S1.
